# Comprehensive RNA-Seq profiling of the lung transcriptome of Bashbay sheep in response to experimental *Mycoplasma ovipneumoniae* infection

**DOI:** 10.1371/journal.pone.0214497

**Published:** 2020-07-08

**Authors:** Zhihui Du, Yanming Sun, Jixue Wang, Haiyan Liu, Yi Yang, Ning Zhao

**Affiliations:** College of Animal Science and Technology, Shihezi University, Shihezi, Xinjiang, China; Clemson University, UNITED STATES

## Abstract

The Bashbay sheep (*Ovis aries*), an indigenous breed of Xinjiang, China, has many excellent characteristics. It is resistant to *Mycoplasma ovipneumoniae* infection, the causative agent of mycoplasma ovipneumonia, a chronic respiratory disease that is harmful to the sheep industry. To date, knowledge regarding the mechanisms responsible for *M*. *ovipneumoniae* pathogenesis in scant. Herein, we report the results of transcriptome profiling of lung tissues from Bashbay sheep experimentally infected with an *M*. *ovipneumoniae* strain at 4 and 14 days post-infection, in comparison to mock-infected animals (0 d). Transcriptome profiling was performed by deep RNA sequencing, using the Illumina platform. The analysis of differentially expressed genes was performed to determine concomitant gene-specific temporal patterns of mRNA expression in the lungs after *M*. *ovipneumoniae* infection. We found 1048 differentially expressed genes (575 up-regulated, 473 down-regulated) when comparing transcriptomic data at 4 and 0 days post-infection, and 2823 (1362 up-regulated, 1461 down-regulated) when comparing 14 versus 0 days post-infection. Kyoto Encyclopedia of Genes and Genomes pathway analysis showed that the differentially expressed genes at 4 and 14 versus 0 days post-infection were enriched in 245 and 287 pathways, respectively, and the Toll-like receptor (TLR) signaling pathway was considered most closely related to MO infection (p < 0.01). Two pathways (LAMP-TLR2/TLR6-MyD88-MKK6-AP1-IL1B and LAMP-TLR8MyD88-IRF5-RANTES) were identified based on the TLR signaling pathway from differentially expressed genes related *M*. *ovipneumoniae* infection. Gene Ontology analysis showed that differentially expressed genes in different groups were enriched for 1580 and 4561 terms, where those most closely related to *M*. *ovipneumoniae* infection are positive regulators of inflammatory responses (p < 0.01). These results could aid in understanding how *M*. *ovipneumoniae* infection progresses in the lungs and may provide useful information regarding key regulatory pathways.

## Introduction

Mycoplasma ovipneumonia, also known as sheep contagious pleuropneumonia, is an infectious disease caused by *Mycoplasma ovipneumoniae* (MO) that not only affects sheep and goats worldwide, but also wild animals, such as bighorn sheep [1–4]. The disease is a highly contagious respiratory disease, characterized by coughing, gasping, progressive weight loss, and pulmonary interstitial hyperplasia inflammation [5]. MO has a higher infection and lethality rate in sheep which are 1–3 months old. Sheep show differing susceptibilities different types of MO infection, and sheep are more susceptible than goats. In some sheep farms, the incidence of MO infection is approximately 2–10%, and the mortality rate in infected sheep can be as high as 100%. Growth of sheep with mycoplasma pneumonia is slow because of severe damage to the lungs, and the feed-conversion ratio of such sheep is significantly higher. Sheep represent the most dangerous source of infection because MO can remain in the lungs for a considerable period of time, and MO can easily spread to the surrounding environment and infect other sheep. Moreover, infection with *MO* also makes sheep more vulnerable to other pathogens [6, 7].

Bashbay sheep (*Ovis aries*) is an indigenous breed of Yumin County (Xinjiang, China) and that many excellent characteristics, such as fast growth and development, high meat production, and disease resistance [8]. The survey conducted by Yan et al. regarding *M*. *ovipneumoniae* infection of sheep in Xinjiang showed that the incidence of mycoplasma ovipneumonia was very low in Bashbay sheep [9]. Bashbay sheep are known to have some susceptibility to MO infection, based on the findings of Du et al. that Bashbay lambs and Argali crossbred lambs could be successfully infected with MO [10]. However, while Argali crossbred lambs infected with MO presented serious clinical symptoms and typical pathological changes with a mortality rate of 33%, the clinical symptoms in Bashbay lambs were mild and the lambs showed fast recovery.

Although the host pathogenesis of MO infection has been a focus of previous research, the entire systematic mechanism of the interaction between MO and the host is unclear. In this study, we achieved our aims of performing the first transcriptomic analysis of sheep infected with MO, using high-throughput sequencing, and determining related immune-inflammatory responses in infected sheep. Therefore, in order to further understand the interaction mechanism between MO and host and to screen differentially expressed genes of Bashbay sheep anti-MO. In this study, RNASeq technology was used to study the transcriptomics of lung tissues of Bashbay sheep infected with MO.

## Material and methods

### MO inoculum and mycoplasma pneumonia disease model

The MO strain was provided by Prof. Yan Genqiang (Preventive Veterinary Laboratory, Animal Science and Technology Institute, Shihezi University, China). The nucleotide sequence of this strain showed 98% homology with the standard Y-98 strain. The experimental procedures were approved by the Laboratory Animal Ethics Committee of the First Affiliated Hospital of Shihezi University School of Medicine (approval number: A2014-079-03). The animals were housed in the stables at the Animal Hospital, College of Animal Science and Technology, Shihezi University and all procedures were carried out in accordance with the institutional ethics committee. The sheep were anaesthetized by Thiophene sodium via the intravenous injection and killed by bloodletting. Nine male Bashbay lambs were obtained from a sheep farm in Yumin County, Xinjiang, China. All the lambs were 2–3 months old, weighed 10–15 kg, and were confirmed to be sero-negative for MO using a commercial enzyme-linked immunosorbent assay (ELISA) kit (R&D Systems, USA).

The MO culture and experimental infection were performed according to the method of Jiang et al. [11]. The concentration of MO used for infection was approximately 10^6^ (Color change unit, CCU)/mL, and each experimental sheep in the 4-day (4d) and 14-day (14d) groups was administered 2 mL of the culture through intratracheal injection. Each treatment group included three sheep. The three control sheep in the 0 d group were instead inoculated with normal saline equivalent to that used in the corresponding experimental sheep. The feed of sheep lacked any antibiotics, and standard clinical examinations, such as, monitoring of body temperature and clinical symptoms, were performed daily. The sheep were sacrificed before infection (0-d group), 4 d post-infection (4-DPI group), or 14 d post-infection (14-DPI group). The lung samples were collected, immediately frozen in liquid nitrogen, and transferred to –80°C. The sera of sheep in the 4-DPI group and 14-DPI group were collected and used for ELISA testing with an MO-specific antibody. Nasal swabs from sheep in the 4-DPI and 14-DPI groups were inoculated on MO-specific medium and incubated at 37°C with 5% CO_2_ for 5 days. MO growth caused the culture medium to change from red to yellow. Biochemical tests, including the glucose-fermentation test and Digitalis saponin test, were carried out to confirm the presence of MO [12].

### RNA extraction, library preparation, and sequencing

The MirVana mRNA Isolation Kit (Ambion-1561, USA) was used to isolate total RNA in accordance with the manufacturer's instructions. Total RNA was purified with Agencourt AMPure XP (Beckman Coulter). RNA purity was assessed by electrophoresis on a 1% agarose gel. The concentration and integrity of RNA were determined using a NanoDrop 2000 (Thermo Scientific) and Agilent Bioanalyzer 2100 (Agilent Technologies), respectively. Samples with an RNA-integrity number (RIN) ≥7 were used for subsequent analysis.

Four micrograms of RNA was used for synthesizing cDNA, which was used to construct libraries with the TruSeq Stranded mRNA LT Sample Prep Kit (Illumina, USA) according to the manufacturer’s recommendations. The libraries were sequenced on an Illumina sequencing platform (HiSeq 2500 NGS system), and 125 bp/150 bp paired-end reads were generated. The sequencing reads have been submitted to the European Nucleotide Archive (www.ebi.ac.uk/arrayexpress/) under the accession number E-MTAB-7408.

### Mapping the sequence reads

Raw sequencing reads (raw reads) in fastq format were first processed using the NGS QC Toolkit [13]. Clean reads were obtained by removing reads containing poly-N, adapter sequences, and low-quality sequences from the raw data. All subsequent analyses were based on the high-quality reads. Reference genome and gene model annotation files were downloaded from an online website (ftp://ftp.ncbi.nlm.nih.gov/genomes/all/GCF_000298735.2_Oar_v4.0/GCF_000298735.2_Oar_v4.0_genomic.fna.gz). An index of the reference genome was built using Bowtie v2.2.3 [14] and paired-end clean reads were aligned to the reference genome sequence using TopHat v2.0.12 [15] (http://tophat.cbcb.umd.edu/).

### Differential gene expression and functional analysis

To identify the differentially expressed genes (DEGs), the number of clean reads mapped to each transcript was counted using HTSeq v0.6.1 [16]. The expression of each transcript was quantified by the parameter, fragments per kilobase of exon per million fragments mapped (FPKM), using cufflinks [17]. Differential gene-expression analysis between two conditions/groups was performed using the DESeq R package (1.20.0), which includes estimateSizeFactors and nbinomTest functions. The data were normalized via the estimateSizeFactors function, and then p-values and fold-change values were calculated using the nbinomTest function [18]. DEGs were screened based on a fold-change in expression of >2 or <0.667, and p-values < 0.05 were considered to reflect differential expression. Subsequently, hierarchical cluster analysis of DEGs was performed to explore the expression pattern of genes.

Kyoto Encyclopedia of Genes and Genomes (KEGG) is a database resource for understanding the functions and utilities of biological systems, such as a cell, an organism, or an ecosystem based on molecular information, especially for large-scale molecular datasets generated by genome sequencing and other high-throughput experimental technologies (http://www.genome.jp/kegg/). KEGG pathway-enrichment analysis of DEGs was performed using KOBAS2.0 software [19] (http://kobas.cbi.pku.edu.cn/home.do). Gene Ontology (GO) enrichment analysis of DEGs was implemented using the GOseq R package [20].

### Validation by reverse transcriptase-quantitative real-time PCR (RT-qPCR)

To validate the RNA-Seq results, six DEGs (ACYP2, CXCR3, FAM195A, ISG20, RHOD, and BPIFA1) were chosen for confirmation of expression diversity by real-time PCR (StepOnePlus, ThermoFisher), and glyceraldehyde-3-phosphatedehydrogenase (GAPDH) was used as an internal reference gene for normalization. All quantitative measurements were taken in triplicate. The real-time PCR primers were designed using Beacon Designer 7 (Table 1). The expression levels of target genes were normalized to the reference gene, and relative expression changes were calculated using the comparative 2^ΔΔCT^ method [21]. Statistical analysis (Mann–Whitney U test) of the RT-qPCR data was performed with GraphPad Prism 5 (GraphPad Software, La Jolla, USA). A p-value <0.05 was considered to reflect a statistically significant difference.

**Table 1:** Details of the RT-qPCR assays used to validate the RNA-Seq results.

Accession number*	Gene symbol	Gene name	Sequence (5′→3′)
XM_012130591.1	ACYP2	Acylphosphatase-2	F: CTGAGCAATGTTGGAAGTC R: AGAGTATTCAAGTTTAGAGATGGT
XM_004022179.3	CXCR3	C-X-C motif chemokine receptor 3	F: ATGAGTGAACGCCAAGAG R: GAAGTACAGCAGAAGTAGGT
XM_015103986	FAM195A	MAPK-regulated corepressor interacting protein 2	F: TGGGACTTCGTGCCAATT R: CTAGGAGCAGTTGGTGATC
XM_012152923	ISG20	Interferon-stimulated exonuclease gene 20	F: TGCTGATGATGGTGACAT R: AACTGAACTGAACTGAACTG
XM_012102172	RHOD	Ras homolog family member D	F:CTTGTGGCTCTTCTTAGG R: TTATCATCTTAAACCGTTTACC
NM_001301405.2	BPIFA1	BPI fold-containing family A member 1	F: TCTCTGCTTGATGGATTG R:CTCAGGAAGGACATTATTCA
NM_001190390.1	GAPDH	Glyceraldehyde-3-phosphate dehydrogenase	F: TGCCAAGTATGATGAGAT R: TCAGTGTAGCCTAGAATG

*National Center for Biotechnology Information), Entrez Gene [http://www.ncbi.nlm.nih.gov/sites/entrez?db=gene]

Note: F indicates the forward primer, and R indicates the reverse primer.

## Results

### Establishment of the mycoplasma pneumonia disease model

The ELISA results showed that all the sheep without MO infection were seronegative and that the three sheep infected with MO were seropositive at 14 DPI. Cotton swabs that were dipped in the nasal cavity were inoculated into the MO medium. After 5–6 days, the color of the medium changed from red to yellow. A small portion of the medium was transferred to a slide and Giemsa staining was performed. Spherical and pleomorphic Mycoplasma could be observed under the microscope. The results of biochemical identification (Table 2) confirmed the identity of the observed bacteria as MO, indicating that the disease model of *M*. *pneumoniae* was successfully established. In addition, all three sheep in the 4-d group were seronegative, and no MO could be isolated. Moreover, clinical signs (coughing, wheezing, and increased temperature) were observed in sheep after MO infection. However, the body temperature increased transiently (40–41°C) and returned to normal after 2–3 days. Only one sheep showed a loss of appetite.

**Table 2:** Results of biochemical identification in the 14-DPI group.

	Glucose fermentation	Digitonin test	Arginine hydrolysis	Triphenyltetrazolium chloride method	Hemadsorption test	Hemolysis test
14-DPI	++	++	——	++	++	β

Note: “+” indicates positive and “—”indicates negative.

### Illumina deep sequencing and read mapping

Nine RNA samples from lung tissues of Bashbay sheep were found suitable for sequencing (S1 Table). Over 4.7 × 10^7^ raw sequence reads were obtained with each group (Table 3) and filtered to remove the low-quality data. After quality control, over 4.4 × 10^7^ clean reads were generated.

**Table 3:** Quality of the sequencing data.

Sample	Raw reads	Raw bases	Clean reads	Clean bases	Valid bases (%)	Q30* (%)	GC (%)
badui1	48065526	7209828900	46205382	6924285726	96.04	92.02	49.00
badui2	47073176	7060976400	44728800	6702572538	94.92	90.95	49.50
badui3	47768218	7165232700	45201298	6773122855	94.53	90.65	50.50
4d_B1_F5	58647486	7330935750	57430094	7177065758	97.90	96.08	48.50
4d_B2_F6	56755988	7094498500	55674790	6957773882	98.07	96.30	49.00
4d_B3_F1	60606678	7575834750	59518162	7438071840	98.18	96.34	49.00
B1_14d_F4	59142856	7392857000	57763054	7218612310	97.64	95.72	51.50
B2_14d_F3	61077468	7634683500	60384736	7546774719	98.84	97.56	50.00
B3_14d_F2	52683600	6585450000	50452264	6305151449	95.74	95.60	49.00

*Q30: the percentage of 99.9%-accurate data among all data

The percent of valid bases was ≥94.53% for each sample. The Q30 values were between 90.02 and 97.56%, which met the requirement that the sequence Q30 must be over 90%. The results indicated that the error rates for the sequencing data were within the allowed range. The GC content was between 48.50% and 51.50%, showing that the sequence met the analytical requirements.

High-quality clean reads were mapped to the sheep (*Ovis aries*) genome, and were mainly distributed in the exon regions, followed by the intergenic regions, and intron regions. The range of the total reads that were mapped to the *Ovis aries* reference genome was between 83.35 and 91.93%, which fulfilled the requirement that the rate of all mapped reads should be more than 70% (S2 Table). In addition, 74.19% to 85.49% of the clean reads that were uniquely mapped to the *Ovis aries* reference genome met the requirement that the proportion of uniquely mapped reads should be more than 70%. Finally, the proportion of multiply mapped reads was between 6.43% and 9.16%, which met the criterion that the proportion of multiply mapped reads should be less than 10%. The above alignments of the analytical data indicated that these data were suitable for further analysis of DEGs.

### Analysis of DEGs

**Fig 1:**
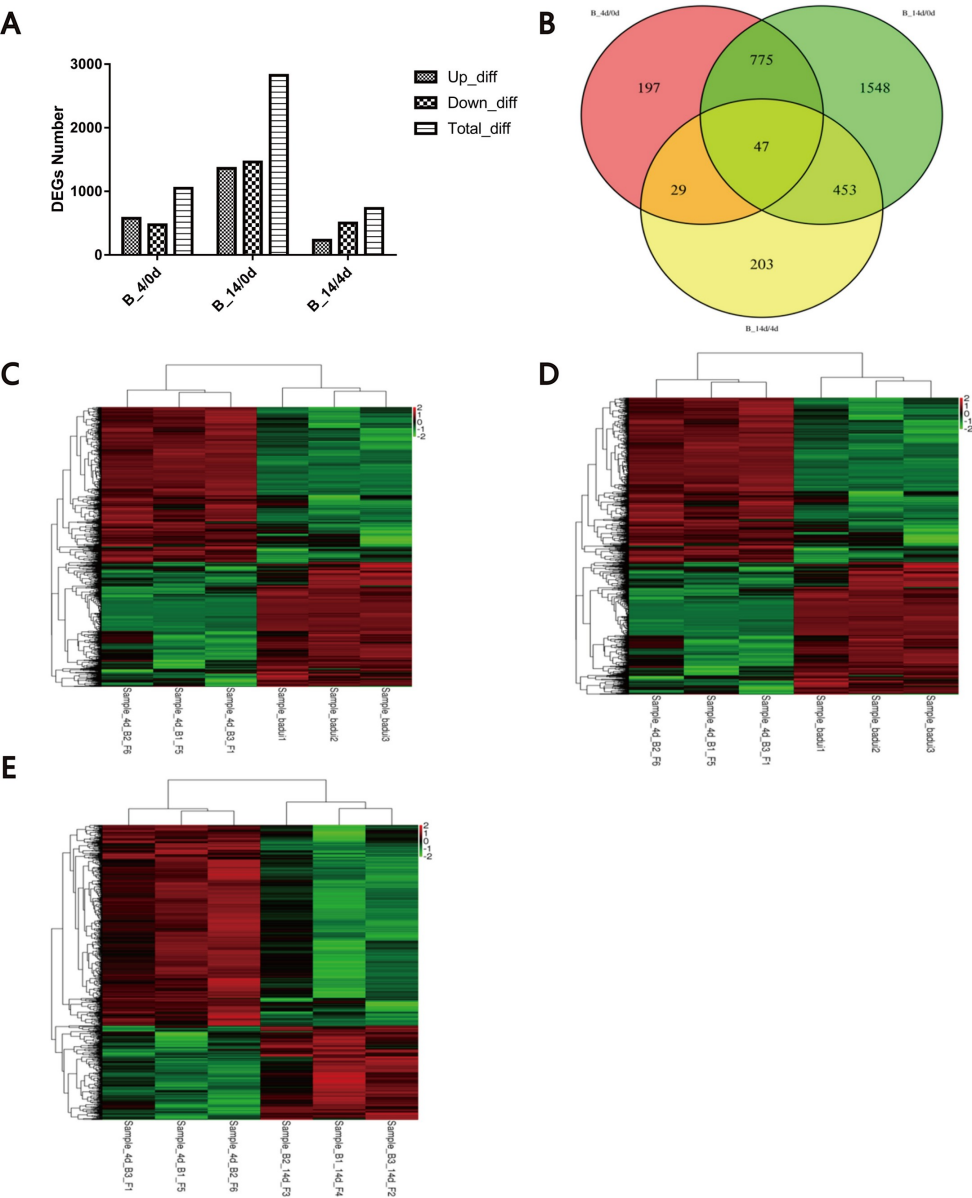
Dynamics of gene expression in Bashbay sheep infected with MO. (A) Number of differentially expressed genes (DEGs) in the host during the infection process. Up, up-regulated DEGs; Down, down-regulated DEGs; Total, total DEGs. (B) Venn diagram of the identified DEGs in the host during the infection process. (C) Hierarchical clustering of DEGs of Bashbay sheep infected with MO in the 4d group as compared to that in the 0d group. The expression levels were visualized using gradient color scheme; the scale from the least abundant to the highest is from -2.0 to 2.0. Green color indicates low expression, and red color indicates high expression of the detected genes. The left vertical axis represents the sample ID. The horizontal axis shows the clusters of samples and the above vertical axis shows the clusters of DEGs. (D) Hierarchical clustering of the DEGs of Bashbay sheep infected with MO in the 14d group as compared to that in the 0d group. (E) Hierarchical clustering of DEGs of Bashbay sheep infected with MO in the 14d group as compared to that in the 4d group.

To better understand the biological mechanisms associated with Bashbay sheep infection with MO, it is critical to identify DEGs at different stages. We identified 1048 DEGs at 4 DPI compared to the 0-d group (B-4/0d comparison), 575 of which were up-regulated and 473 were down-regulated, accounting for 54.87% and 45.13% of the DEGs, respectively (Fig 1A). In addition, 2823 DEGs were detected at 14 DPI compared the 0-d group (B-14/0d comparison), 1362 of which were up-regulated and 1461 were down-regulated, accounting for 48.25% and 51.75% of the DEGs, respectively. We identified 732 DEGs between the 14-DPI and 4-DPI groups (B-14/4d comparison), 232 of which were up-regulated and 500 were down-regulated, accounting for 31.69% and 68.31% of the DEGs, respectively. Furthermore, 14 DEGs were closely related with MO infection, encoding proteins that are directly or indirectly involved in host defenses against pathogens (Table 4).

**Table 4:** DEGs related to MO infection.

Gene symbol	Description	Fold-change		p-value	
		4/0d	14/0d	4/0d	14/0d
MYD88	Myeloid differentiation primary response 88	0.85	1.03	0.04	0.0092
NFKB1	Nuclear factor kappa B subunit 1	0.83	0.89	0.036	0.03
IL1B	Interleukin 1 beta	2.53	4.55	0.043	0.006
IL6	Interleukin 6	1.86	1.66	0.041	0.018
TLR2	Toll-like receptor 2	0.87	9.81	0.0057	0.007
TLR4	Toll-like receptor 4	0.71	0.92	0.06	0.039
TLR6	Toll-like receptor 6	1.68	1.75	0.01	0.03
TLR8	Toll-like receptor 8	1.34	6.41	0.018	0.008
IRAK1	Interleukin 1 receptor-associated kinase 1	1.24	2.01	0.037	0.015
IRAK4	Interleukin 1 receptor-associated kinase 4	1.11	1.24	0.0061	0.027
IRF5	Interferon regulatory factor 5	3.20	6.33	0.042	0.016
CXCL8	Chemokine (C-X-C motif) ligand 8	2.85	19.45	0.002	0.0001
MAP2K6 (MKK6)	Mitogen-activated protein kinase 6	2.14	7.37	0.035	0.023
BPIFA1 (SPLUNC1)	BPI fold-containing family A member 1	1.86	19.72	0.0056	0.025

The DEGs among the different groups are shown in the Venn diagrams (Fig 1B). We found 197, 1548, and 203 unique DEGs by performing the B-4/0d, B-14/0d, and B-14/4d comparisons, respectively. In addition, 822 common DEGs were found between B-4/0d and B-14/0d, 500 common DEGs were found between B-14/0d and B-14/4d, and 76 common DEGs were found between B-4/0d and B-14/4d. Forty-seven DEGs were in common between these groups.

Heatmaps reflecting the expression patterns of host mRNAs were constructed based on the FPKM values. The heatmap representation of DEGs showed that the samples were clustered together at 0 d (badui1, badui2, and badui3), 4 DPI (4d_B1_F5, 4d_B2_F6, and 4d_B3_F1), and 14 DPI (B1_14d_F4, B2_14d_F3, and B3_14d_F2), as shown in Fig 1C–1E. Our data revealed that the samples had a similar transcriptome expression patterns at each stage. Our findings also showed that the physiological status of the selected sheep were similar and that the disease model of *M*. *ovipneumonia* was successfully established.

### KEGG pathway analysis

**Fig 2:**
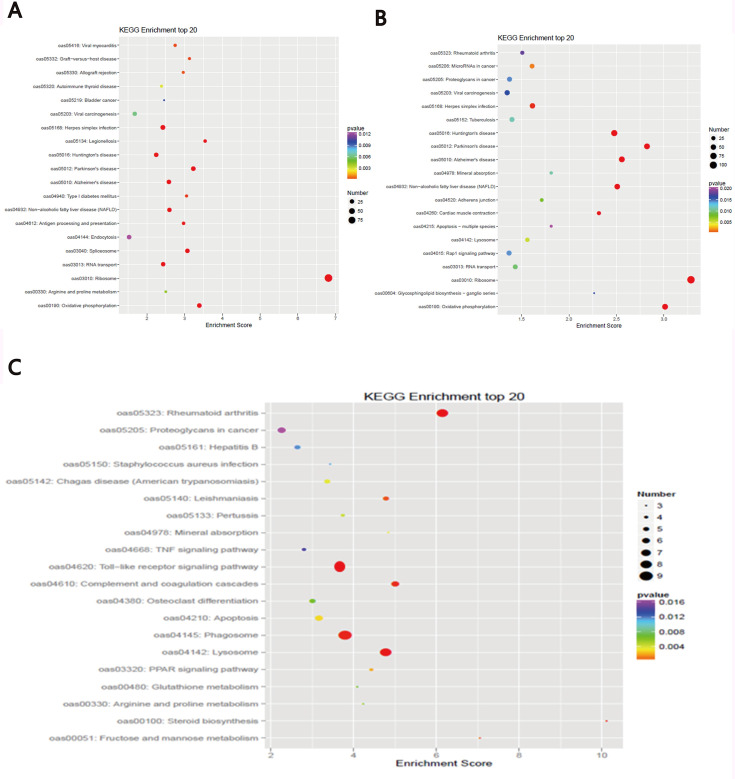
Top 20 KEGG enriched pathways during the infection process. The x-axis shows the enrichment factor. The y-axis corresponds to the KEGG Pathway. The color of the dot represents the p-value and the size of the dot represents the number of DEGs mapped to the reference pathways. (A) Top 20 enriched pathways in B-4/0d. (B) Top 20 enriched pathways in B-14/0d. (C) Top 20 enriched pathways in B-14/4d.

To identify biological pathways operating in sheep infected with MO, the KEGG database was used to analyze the DEGs among the different groups. With the B-4/0d comparison, we identified 245 KEGG enrichment items, the top 20 of which showed that the DEGs of sheep infected with MO were related to “antigen processing and presentation,” “endocytosis,” and “oxidative phosphorylation” (Fig 2A). The B-14/0d comparison revealed 287 KEGG enrichment items, the top 20 of which showed that the significantly enriched pathways were related to “adherens junction,” “focal adhesion,” “Wnt signaling pathway,” “Rap1 signaling pathway,” “Ras signaling pathway,” “phagosome,” and “oxidative phosphorylation” (Fig 2B). Additionally, 236 KEGG enrichment items were found with the B-14/4d comparison, the top 20 of which showed that the significantly enriched pathways included “antigen processing and presentation,” “focal adhesion,” “Toll-like receptor signaling pathway,” “complement and coagulation cascades,” and “PPAR signaling pathway” (Fig 2C).

**Fig 3:**
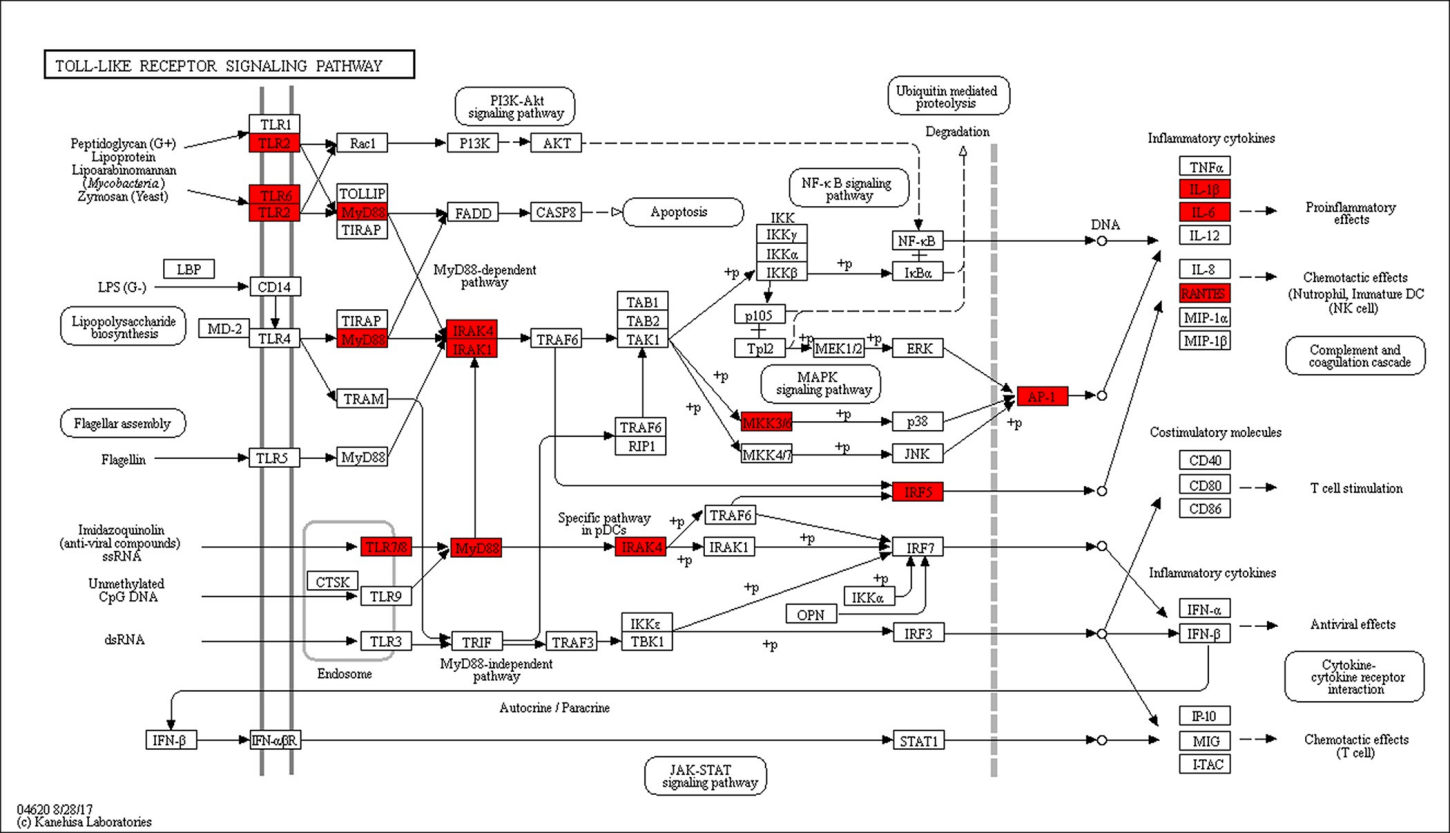
Toll receptor pathway diagram of Bashbay sheep induced by MO. Red: Up-regulated expression genes; Black: Stable expression genes.

The “Toll-like receptor signaling pathway” (p = 2.39 E-17) was the top KEGG enrichment item that was mostly related with MO infection (Table 5). Two small pathways (first: LAMP-TLR2/TLR6-MyD88-MKK6-AP1-IL1B; second: LAMP-TLR8-MyD88-IRF5-RANTES) were identified using the KEGG database, indicating that DEGs related with MO infection were mapped to the TLR signaling pathway (Fig 3).

**Table 5:** The KEGG enrichment items most closely related to MO after Bashbay sheep infected with MO.

Comparison	KEGG ID	KEGG Term	P-value	Number of DEGs
B-4/0d	path:oas00190	Oxidative phosphorylation	1.84E-09	28
	path:oas04612	Antigen processing and presentation	5.44E-05	14
	path:oas04144	Endocytosis	1.23 E-02	25
B-14/0d	path:oas00190	Oxidative phosphorylation	4.43 E-03	59
	path:oas04142	Lysosome	5.20 E-03	28
	path:oas04215	Apoptosis—multiple species	2.04 E-02	9
B-14/4d	path:oas04142	Lysosome	4.18 E-05	8
	path:oas04145	Phagosome	1.21 E-04	9
	path:oas04610	Complement and coagulation cascades	1.84 E-04	6
	path:oas04620	Toll-like receptor signaling pathway	2.39 E-17	14

### GO enrichment analysis

**Fig 4:**
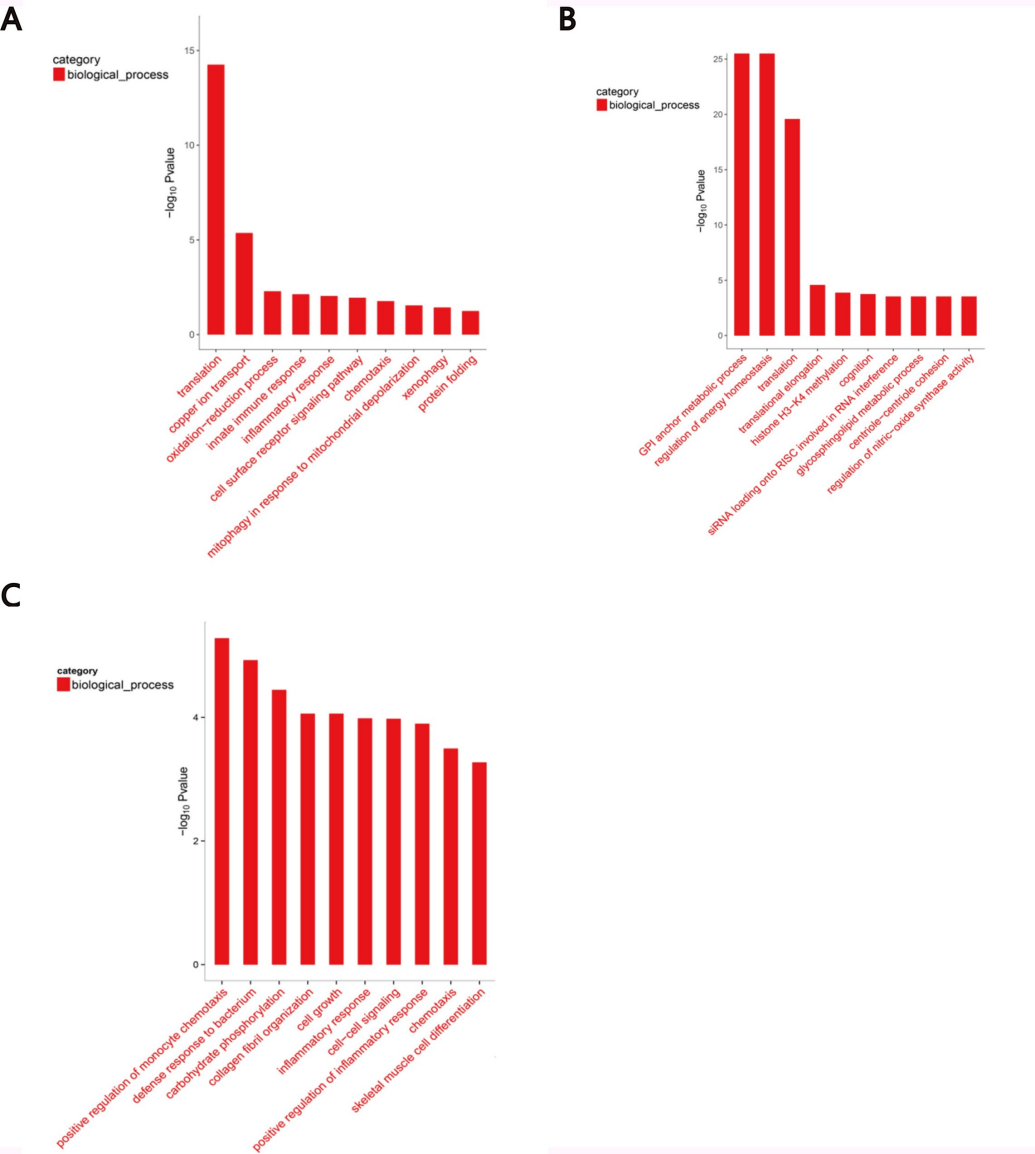
Top 10 enriched gene ontology (GO) biological processes in Bashbay sheep infected with MO. These differentially expressed genes detected by RNA-Seq were investigated for significantly enriched biological processes (p < 0.01). The left vertical axis represents significance. The horizontal axis shows the GO terms. (A) Top 10 GO terms in B-4/0d. (B) Top 10 GO terms in B-14/0d. (C) Top 10 GO terms in B-14/4d.

GO enrichment analysis, which is an international standardized gene-function classification system, includes three major functional categories, namely biological processes, cellular components, and molecular functions. GO analysis was performed to detect significantly enriched GO terms for the DEGs. In our study, 1580 GO terms were found when performing the B-4/0d comparison, including 944 biological process terms (59.75%), 395 molecular function terms (25.00%), and 304 cellular component terms (15.25%). We found 4561 GO terms when performing the B-14/0d comparison, which included 3068 biological process terms (67.27%), 969 molecular function terms (21.25%), and 524 cellular component terms (11.48%). We found 833 GO terms were performing the B-14/4d comparison, which included 517 biological process terms (62.06%), 218 molecular function terms (26.17%), and 98 cellular component terms (11.77%). Moreover, the top 10 GO terms for biological processes included “oxidation–reduction process,” “innate immune response,” “inflammatory response,” and “cell surface receptor signaling pathway” with B-4/0d (Fig 4A). The top 10 GO terms for biological processes included “regulation of energy homeostasis,” “siRNA loading on RISC involved in RNA interference,” and “regulation of nitric-oxide synthase activity” with B-14/0d (Fig 4B). The top 10 GO terms for biological processes included “positive regulation of monocyte chemotaxis,” “defense response to bacterium,” “positive regulation of inflammatory response,” and “cell growth” with B-14/4d (Fig 4C).

The main functional descriptions of GO terms significant enriched for DEGs associated with MO infection in Bashbay sheep were inflammatory responses (p = 9.15E-04), innate immune responses (p = 7.42E-06), and positive regulation of inflammatory response (p = 1.27E-07) (Table 6). The correlation with the innate immune response was higher than with inflammatory reaction at 4 DPI than at 0 DPI, while that of inflammatory reaction decreased at 14 DPI compared to 4 DPI. The most closely related GO term was positive regulation of inflammatory response.

**Table 6:** The most notable differentially expressed GO enrichment items closely related to MO infection in Bashbay sheep.

Comparison	GO ID	GO Term	P-value	Number of DEGs
B-4/0d	GO:0055114	Oxidation-reduction process	5.17 E-03	16
	GO:0045087	Innate immune response	7.42 E-06	4
	GO:0006954	Inflammatory response	9.15 E-04	5
	GO:0007166	Cell surface receptor signaling pathway	1.14 E-02	4
	GO:0006935	Chemotaxis	1.71 E-02	3
B-14/0d	GO:0050890	Cognition	1.77 E-04	8
	GO:0006687	Glycosphingolipid metabolic process	2.93 E-04	3
	GO:0050999	Regulation of nitric-oxide synthase activity	2.93 E-04	3
B-14/4d	GO:0090026	Positive regulation of monocyte chemotaxis	5.26 E-06	3
	GO:0042742	Defense response to bacterium	1.19 E-05	5
	GO:0006954	Inflammatory response	1.04 E-03	6
	GO:0007267	Cell-cell signaling	1.06 E-04	3
	GO:0050729	Positive regulation of inflammatory response	1.27 E-07	3
	GO:0006935	Chemotaxis	3.20 E-04	4

### Confirmation of RNA-Seq data by RT-qPCR

**Fig 5:**
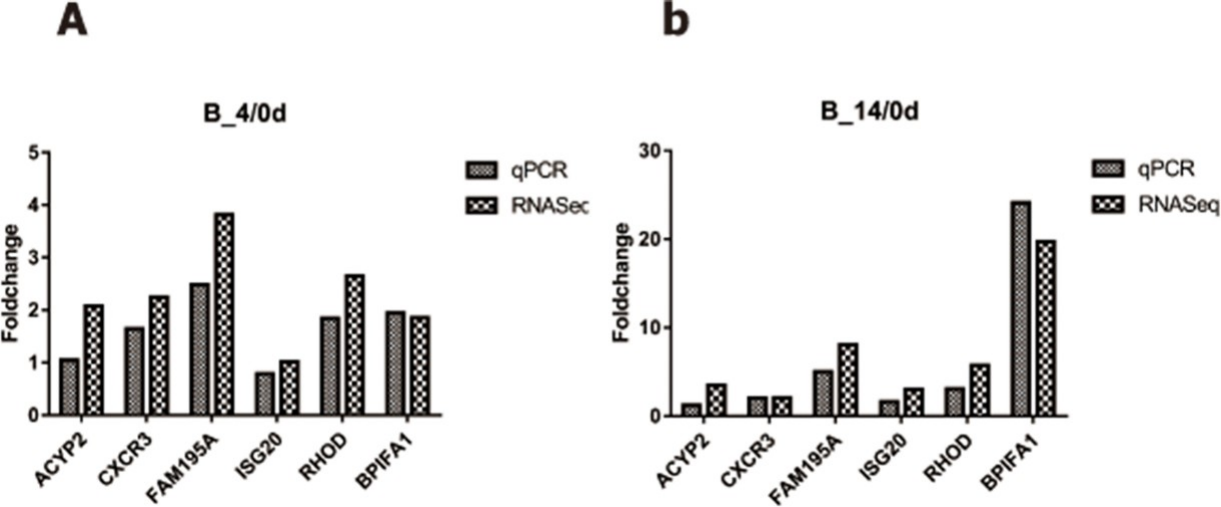
Validation of RNA-seq results by real-time polymerase chain reaction. (A) Validation of RNA-Seq results by real-time PCR in B-4/0d. (B) Validation of RNA-Seq results by real-time PCR in B-14/0d.

To validate the sequencing results, six DEGs (ACYP2, CXCR3, FAM195A, ISG20, RHOD, and BPIFA1) and an internal reference gene (GAPDH) were selected for real-time qPCR analysis. The results of real-time PCR of all the selected genes were in good agreement with the RNA-Seq data, indicating the accuracy and reliability of the RNA-Seq data obtained in this study (Fig 5).

## Discussion

In this study, Bashbay sheep were infected with MO, after which MO antibodies were detected in three sheep at 14 DPI and MO was isolated from nasal swabs. Furthermore, biochemical identification results confirmed the biochemical characteristics of MO. These results indicated that the experimental MO infection was successful. In addition, all three sheep in the 4-d group were seronegative, and MO was not isolated from nasal swabs. As reported by Yan et al., this outcome could be explained by the fact that the host did not produce antibodies until 2 weeks after MO infection and did not excrete the etiological agent until 6 DPI [9]. Clinical observations showed that cough and wheezing occurred, and the body temperature increased transiently (40–41°C) in sheep in the 4-DPI and 14-DPI groups. The sheep in the 4-DPI group showed signs of disease (temperature and coughing), but were not positive for MO antibodies and MO was not isolated.

The mechanism of mycoplasma pathogenesis in host is still insufficient. Currently, pathology is thought to mainly stem from damage to the host immune response, caused by mycoplasma infection [22–24]. Some studies have shown that a series of inflammatory reactions are caused by mycoplasma infections, owing to their lipid-associated membrane proteins (LAMPs) [25–27]. LAMPs may mediate mycoplasma adhesion to the host cell surface, thereby facilitating subsequent host cell entry, leading to the host cell damage and death [28]. Furthermore, most mycoplasma lipoproteins are exposed on its surface. Some lipoproteins and cytosolic proteins of gram-negative bacteria are virulent [29] or the target of antibodies [30]. Therefore, they can affect the host immune system by continuously interacting with cells in the host’s body [31].

It has been reported that LAMPs of *M*. *fermentans* (Mfe) or their derivative, macrophage-activating lipopeptide-2 (MALP-2), could interact with Toll-like receptors (TLRs) on the surface of monocytes/macrophages and affect the immune response of the host [32]. In addition, they elicit inflammatory reactions by activating nuclear factor kappa B (NF-κB) and activator protein-1 (AP-1), mediated by myeloid differentiation factor 88 (MyD88) and Fas-related death domains. Thus, they can induce a variety of proinflammatory cytokines and bioactive substances that are synthesized and secreted by mononuclear macrophages, such as TNF-α, IL-1β, and IL-6, reactive oxygen species, lysosomal enzymes, and nitric oxide [33–37]. LAMPs are also capable of inducing apoptosis by activating p38 mitogen-activated protein kinases (MAPKs), myeloid differentiation factor 88 (MyD88), and other signal-transduction pathways [38,39].

Previous data have shown that TLRs play a crucial role against *M*. *ovipneumoniae* infection [40]. It has been reported that mycoplasma are recognized by TLR2, TLR2/TLR4, TLR2/TLR6, and TLR2/TLR4/TLR6 [41–44]. Some mycoplasma components, such as the 19-kDa mycobacterial lipoprotein and lipid-associated membrane proteins, are recognized by TLR2 on the surface of macrophages, which secrete cytokines to promote activation of the host immune response. Xiao et al. [45] found that TLR2 and TLR4 were up-regulated in pigs at 28 DPI with *M*. *pneumoniae*. Yoshihiro [46] infected porcine alveolar macrophages with *M*. *hyopneumoniae in vitro* and found that TLR2 and TLR6 were up-regulated at 48 h after infection. In the present study, TLR2 was up-regulated in B-14/0d (9.81-fold), TLR6 was up-regulated in B-4/0d (1.68-fold) and B-14/0d (1.75-fold), and TLR8 was up-regulated in B-4/0d (1.34-fold) and in B-14/0d (6.41-fold). These results were consistent with those of Liu Xiao and Yoshihiro. However, TLR2 and TLR4 were down-regulated in B-4/0d (0.87 and 0.71-fold, respectively) and TLR4 was down-regulated in B-14/0d (0.92-fold); these results were different from those of Xiao et al. [45]. This discrepancy could have been caused by different mycoplasma strains and host cells, the manner of stimulation, and the duration of infection.

KEGG analysis of Bashbay sheep infected with MO revealed that 14 DEGs were enriched in the TLR signaling pathway (Fig 2C), including IRF7, LOC101114535, CCL5, MAPK10, MAPK8, PIK3R1, FOS, TLR2, TLR4, TLR6, MYD88, IRF5, and MAP2K6 (mkk6). MyD88 is one of the most important adaptors in innate immune response-signal pathways mediated by TLRs. The activation and inactivation of several downstream signals related to immunity depend on MyD88 activity [40,47,48]. Data from Lai et al. showed that macrophage accumulation and activation decreased in the lungs of MyD88(−/−) mice and *Mycoplasma pneumoniae* (Mp) clearance was impaired, indicating that MyD88 is a key signaling protein in the anti-Mp response [49]. Most TLRs can mediate MyD88 activation and induce the activation of transforming growth factor-activated kinase 1 (TAK1) and mitogen-activated protein kinase kinase (MKK6), and subsequently activate the MAPK p38 signaling pathway, which leads to the secretion of IL-1β and other proinflammatory cytokines [50]. The DEG data obtained in the present study showed that MYD88 was down-regulated in B-4/0d (0.85-fold) and was up-regulated in B-14/0d (1.03-fold); MAP2K6 (MKK6) was up-regulated in B-4/0d (2.14-fold) and in B-14/0d (7.37-fold); IL-1β was up-regulated in B-4/0d (2.53-fold) and in B-14/0d (4.55-fold). These results indicate that MO infection should transduce signals and stimulate the host to produce the corresponding cytokines via the LAMP-TLR2/TLR6-MyD88-MKK6-AP1-IL1B pathway. TLRs are pattern-recognition receptors that play important role in early innate immune recognition and host inflammatory response to invasive microorganisms. Gally et al. previously showed that intact TLR2 signaling is critical for host defense-related cytokine production and Mp clearance [51]. Activation of this signal pathway indicates that MO lipoproteins can stimulate TLR2 production in Bashbay sheep, which can induce host production of the defensive cytokine IL-1 and promote MO clearance. This may explain why Bashbay sheep are resistant to MO infection. These results could be helpful in understanding how MO infection progresses in the lung and provide information on key pathways that regulate it.

Several interferon-regulatory factors (IRFs), such as IRF1, IRF3, IRF5, IRF7, and IRF8, are activated downstream of some signaling pathways. In particular, IRF5 regulates the MyD88-dependent TLRs signaling pathway in terms of inflammatory cytokines, which plays a crucial role in promoting inflammatory macrophage polarization and RANTES secretion [52–56]. In the present study, IRF5 was up-regulated in B-4/0d (3.20-fold) and B-14/0d (6.33-fold), and IRF7 was up-regulated in B-4/0d (1.50-fold) and B-14/0d (3.09-fold). The term “phagocytosis” was also significantly enriched in the KEGG analysis (Fig 2B). Therefore, another signal-transduction (i.e., the LAMP-TLR8-MyD88-IRF5-RANTES pathway) is important for inducing host cell RANTES secretion, which initiates macrophage phagocytosis of MO.

The stimulation of human lung epithelial NCI-H292 cells with a TLR2 agonist has been reported to increase SPLUNC1 expression in NCI-H292 cells in dose- and time-dependent manners, and to enhance the promoter activity of SPLUNC1 [57]. Gally et al. used gene-knockout technology to produce SPLUNC1 gene-deficient mice [58]. The levels of Mp in SPLUNC1−/− mice increased by three times compared to the levels in wild-type control mice upon infection with Mp. In contrast, mice overexpressing hSPLUNC1 exclusively in airway epithelial cells had three times less Mp compared to that in the control group. These findings demonstrate that SPLUNC1 is essential and plays a vital role *in vivo* in host defense against mycoplasma infection. In the present study, SPLUNC1 was up-regulated in B-4/0d (1.86-fold) and in B-14/0d (19.72-fold). Moreover, SPLUNC1 expression increased further at later times post-infection.

**Fig 6:**
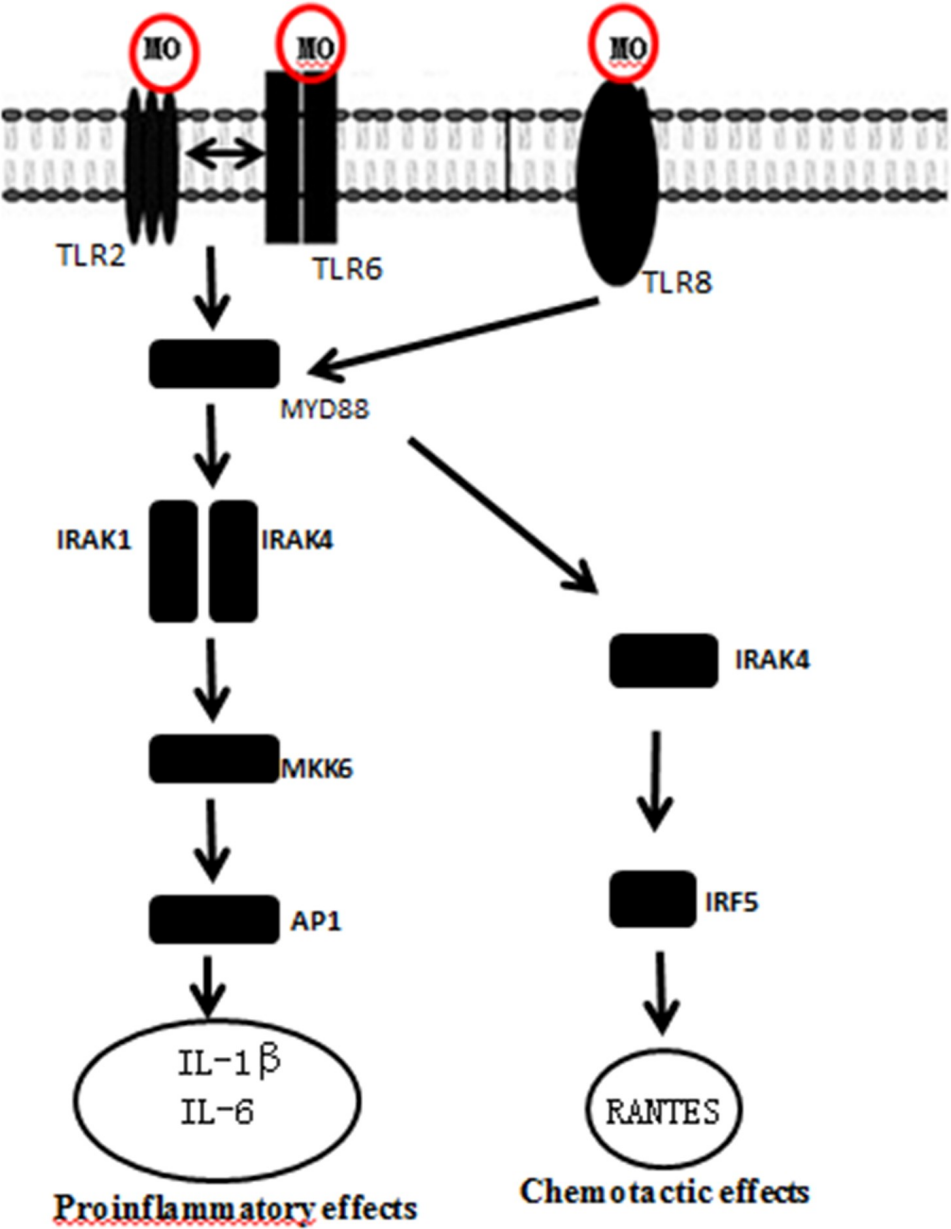
Two small pathways in toll receptor pathway induced by MO.

In summary, we report the results of the comprehensive transcriptomic analysis of Bashbay sheep infected with MO. At 4 or 14 days post-MO infection, two main signal transduction pathways were induced, which stimulated the host to produce the corresponding cytokines (Fig 6). The first signaling pathway was the LAMP-TLR2/TLR6-MyD88-MKK6-AP1-IL1B pathway and the second pathway was the LAMP-TLR8-MyD88-IRF5-RANTES pathway. Our findings should provide a reference for better understanding host–pathogen-interaction mechanisms in general.

## Supporting information

S1 Table RNA integrity numbers of the different samples. (DOCX)

S2 Table Percent of reads uniquely mapped to reference genomes compared to total mapped. (DOCX)
