# Supplementary material for: Comprehensive RNA-Seq profiling of the lung transcriptome of Bashbay sheep in response to experimental Mycoplasma ovipneumoniae infection
Source: PLoS One. 2020 Jul 8;15(7):e0214497. doi: 10.1371/journal.pone.0214497 (PMC7343132; doi:10.1371/journal.pone.0214497)
Supplement: S1 Table — (DOCX) [file pone.0214497.s001.docx]

| **S1 Table RNA integrity numbers of the different samples** | | | | |
| --- | --- | --- | --- | --- |
| **Sample** | **A260/280** | **RIN** | **Result** |  |
| badui1 | 2.12 | 8.8 | OK |  |
| badui2 | 2.13 | 8.7 | OK |  |
| badui3 | 2.09 | 8.3 | OK |  |
| 4d B1 F5 | 2.11 | 8.2 | OK |  |
| 4d B2 F6 | 2.10 | 9.2 | OK |  |
| 4d B3 F1 | 2.10 | 7.8 | OK |  |
| B1 14d F4 | 2.10 | 7.0 | OK |  |
| B2 14d F3 | 2.11 | 7.1 | OK |  |
| B3 14d F2 | 2.10 | 7.0 | OK |  |
