# Supplementary material for: Comprehensive RNA-Seq profiling of the lung transcriptome of Bashbay sheep in response to experimental Mycoplasma ovipneumoniae infection
Source: PLoS One. 2020 Jul 8;15(7):e0214497. doi: 10.1371/journal.pone.0214497 (PMC7343132; doi:10.1371/journal.pone.0214497)
Supplement: S2 Table — (DOCX) [file pone.0214497.s002.docx]

| **S2 Table Percent of reads uniquely mapped to reference genomes compared to total mapped** | | | | | | | | | | | | |
| --- | --- | --- | --- | --- | --- | --- | --- | --- | --- | --- | --- | --- |
| Sample | Total reads | | Total mapped | Multiple  Mapped | Uniquely  Mapped | Read-1 | Read-2 | Reads map to‘+’ | Reads map to‘-’ | Non-splice reads | Splice reads | Reads mapped in proper pairs |
| Sample_badui1 | 46205382 | 40569358(87.80%) | | 2991492(6.74%) | 37577866(81.33%) | 18818852(40.73%) | 18759014(40.60%) | 18782010(40.65%) | 18795856(40.68%) | 23273171(50.37%) | 14304695(30.96%) | 35350766(76.51%) |
| Sample _badui2 | 44728800 | 40928017(91.50%) | | 3008351(6.73%) | 37919666(84.78%) | 19016384(42.51%) | 18903282(42.26%) | 18962547(42.39%) | 18957119(42.38%) | 23389033(52.29%) | 14530633(32.49%) | 36260648(81.07%) |
| Sample_badui3 | 45201298 | 41552259(91.93%) | | 2907723(6.43%) | 38644536(85.49%) | 19403136(42.93%) | 19241400(42.57%) | 19316771(42.73%) | 19327765(42.76%) | 23715220(52.47%) | 14929316(33.03%) | 36906344(81.65%) |
| Sample_4d_B1_F5 | 57430094 | 49580243(86.33%) | | 4712065(8.20%) | 44868178(78.13%) | 22929421(39.93%) | 21938757(38.20%) | 22470927(39.13%) | 22397251(39.00%) | 32802754(57.12) | 12065424(21.01%) | 37643458(65.55%) |
| Sample_4d_B2_F6 | 55674790 | 47603541(85.50%) | | 4365114(7.84%) | 43238427(77.66%) | 22077727(39.65%) | 21160700(38.01%) | 21642288(38.87%) | 21596139(38.79%) | 29865988(53.64%) | 13372439(24.02%) | 36134558(64.90%) |
| Sample_4d_B3_F1 | 59518162 | 50768617(85.30%) | | 5203747(8.74%) | 45564870(76.56%) | 23268646(39.10%) | 22296224(37.46%) | 22819361(38.34%) | 22745509(38.22%) | 32058469(53.86%) | 13506401(22.69%) | 38246840(64.26%) |
| Sample_B1_14d _F4 | 57763054 | 48146359(83.35%) | | 5292801(9.16%) | 42853558(74.19%) | 21988362(38.07%) | 20865196(36.12%) | 21440401(37.12%) | 21413157(37.07%) | 28867184(49.98%) | 13986374(24.21%) | 35510848(61.48%) |
| Sample_B2_14d _F3 | 60384736 | 52076881(86.24%) | | 5516975(9.14%) | 46559906(77.11%) | 23437117(38.81%) | 23122789(38.29%) | 23316968(38.61%) | 23242938(38.49%) | 32604814(54.00%) | 13955092(23.11%) | 39049230(64.67%) |
| Sample_B3_14d _F2 | 50452264 | 43234054(85.69%) | | 4364953(8.65%) | 38869101(77.04%) | 19682332(39.01%) | 19186769(38.03%) | 19474496(38.60%) | 19394605(38.44%) | 27578830(54.66%) | 11290271(22.38%) | 32809316(65.03%) |
